# Supplementary material for: A natural catastrophic turnover event: individual sociality matters despite community resilience in wild house mice
Source: Proc Biol Sci. 2020 May 6;287(1926):20192880. doi: 10.1098/rspb.2019.2880 (PMC7282912; doi:10.1098/rspb.2019.2880)
Supplement: Supplementary Tables and Figures [file rspb20192880supp1.pdf]

Supplementary table 1: Model results for the difference in individuals' degree centrality in survivor-only networks (Figure 2 a, c to f) from the survivor-only pre-event network (Figure 2 b). Explanatory variables are an individual's level of association with missing individuals, pre-event sociality(weighted degree in the full pre-event network), timestep and the interaction between them. p values obtained by comparisons with randomised versions of the survivor-only post-event networks.

	Estimate	l-95% CI	u-95% CI	Mean rand. est.	SD rand. est.	p-value
Intercept	0.02	-0.76	0.83	0.08	0.08	0.24
Timestep 1 (-10 to -6 days)	-0.41	-1.41	0.60	-1.99	0.96	<0.05
Timestep 3 (1 to 5 days)	5.27	4.24	6.26	4.99	0.69	0.33
Timestep 4 (6 to 10 days)	-0.25	-1.24	0.73	-1.80	0.37	<0.05
Timestep 5 (11 to 15 days)	-2.43	-3.45	-1.48	-3.03	0.28	<0.05
Timestep 6 (16 to 20 days)	-2.75	-3.77	-1.80	-2.89	0.25	0.29
Prop. missing	0.11	-0.82	1.03	0.33	0.29	0.2
Sociality	-0.13	-0.91	0.66	-0.07	0.10	0.26
Timestep 1 (-10 to -6 days) : Prop. missing	-0.49	-1.64	0.61	0.78	0.89	<0.05
Timestep 3 (1 to 5 days) : Prop. missing	1.05	-0.10	2.22	0.44	0.96	0.26
Timestep 4 (6 to 10 days) : Prop. missing	-0.60	-1.76	0.52	0.76	0.77	<0.05
Timestep 5 (11 to 15 days) : Prop. missing	-0.98	-2.11	0.18	0.96	0.76	<0.05
Timestep 6 (16 to 20 days) : Prop. missing	-1.87	-2.99	-0.77	0.77	0.84	<0.05
Timestep 1 (-10 to -6 days) : Sociality	-0.26	-1.24	0.71	-3.05	0.75	<0.05
Timestep 3 (1 to 5 days) : Sociality	-0.66	-1.62	0.33	-2.85	0.97	<0.05
Timestep 4 (6 to 10 days) : Sociality	-1.21	-2.22	-0.22	-3.17	0.59	<0.05
Timestep 5 (11 to 15 days) : Sociality	-0.50	-1.47	0.48	-3.16	0.56	<0.05
Timestep 6 (16 to 20 days) : Sociality	-0.34	-1.29	0.67	-3.03	0.53	<0.05
Prop. missing : Sociality	0.11	-0.76	1.02	0.33	0.34	0.22
Timestep 1 (-10 to -6 days) : Prop. missing : Sociality	-0.08	-1.18	1.03	-2.66	0.97	<0.05
Timestep 3 (1 to 5 days) : Prop. missing : Sociality	-0.17	-1.26	0.92	-3.00	1.00	<0.05
Timestep 4 (6 to 10 days) : Prop. missing : Sociality	-0.95	-1.98	0.11	-2.74	0.88	<0.05
Timestep 5 (11 to 15 days) : Prop. missing : Sociality	-1.47	-2.57	-0.38	-2.66	0.87	<0.05
Timestep 6 (16 to 20 days) : Prop. missing : Sociality	-1.86	-2.99	-0.77	-2.71	0.93	<0.05

Variance estimates:

	Estimate	l-95% CI	u-95% CI	Mean rand. est.	SD rand. est.	p-value
Intercept	1.63	1.59	1.67	2.34	0.04	<0.05
Prop. missing	0.18	0.14	0.23	0.02	0.03	<0.05
Sociality	0.03	-0.01	0.07	-0.01	0.03	<0.05
Prop. missing : Sociality	0.19	0.15	0.23	0.05	0.04	<0.05

Model results for the difference in individuals' weighted degree centrality in survivor-only networks (Figure 2 a, c to f) from the survivor-only pre-event network (Figure 2 b). Explanatory variables are an individual's level of association with missing individuals, pre-event sociality(weighted degree in the full pre-event network), timestep and the interaction between them. p values obtained by comparisons with randomised versions of the survivor-only post-event networks.

	Estimate	l-95% CI	u-95% CI	Mean rand. est.	SD rand. est.	p-value
Intercept	0.02	-0.11	0.15	0.00	0.00	<0.05
Timestep 1 (-10 to -6 days)	-0.83	-0.97	-0.68	-1.09	0.06	<0.05
Timestep 3 (1 to 5 days)	0.11	-0.05	0.26	-0.24	0.07	<0.05
Timestep 4 (6 to 10 days)	-0.34	-0.50	-0.19	-0.65	0.06	<0.05
Timestep 5 (11 to 15 days)	0.42	0.27	0.56	0.37	0.05	<0.05
Timestep 6 (16 to 20 days)	0.04	-0.11	0.19	0.06	0.04	0.28
Prop. missing	0.05	-0.08	0.19	0.00	0.00	<0.05
Sociality	-0.01	-0.14	0.11	0.00	0.00	<0.05
Timestep 1 (-10 to -6 days) : Prop. missing	0.03	-0.14	0.19	0.84	0.05	<0.05
Timestep 3 (1 to 5 days) : Prop. missing	0.07	-0.10	0.24	0.81	0.10	<0.05
Timestep 4 (6 to 10 days) : Prop. missing	0.09	-0.08	0.26	0.85	0.08	<0.05
Timestep 5 (11 to 15 days) : Prop. missing	-0.01	-0.18	0.16	0.84	0.12	<0.05
Timestep 6 (16 to 20 days) : Prop. missing	-0.02	-0.19	0.14	0.84	0.12	<0.05
Timestep 1 (-10 to -6 days) : Sociality	-0.44	-0.59	-0.29	-1.01	0.05	<0.05
Timestep 3 (1 to 5 days) : Sociality	-0.42	-0.57	-0.27	-1.04	0.11	<0.05
Timestep 4 (6 to 10 days) : Sociality	-0.36	-0.50	-0.21	-1.04	0.10	<0.05
Timestep 5 (11 to 15 days) : Sociality	-0.17	-0.32	-0.02	-1.02	0.11	<0.05
Timestep 6 (16 to 20 days) : Sociality	-0.21	-0.35	-0.06	-0.97	0.12	<0.05
Prop. missing : Sociality	0.06	-0.08	0.20	0.00	0.00	<0.05
Timestep 1 (-10 to -6 days) : Prop. missing : Sociality	-0.06	-0.22	0.10	0.27	0.06	<0.05
Timestep 3 (1 to 5 days) : Prop. missing : Sociality	-0.14	-0.31	0.03	0.25	0.10	<0.05
Timestep 4 (6 to 10 days) : Prop. missing : Sociality	-0.14	-0.31	0.02	0.27	0.09	<0.05
Timestep 5 (11 to 15 days) : Prop. missing : Sociality	-0.07	-0.25	0.10	0.26	0.12	<0.05
Timestep 6 (16 to 20 days) : Prop. missing : Sociality	-0.02	-0.19	0.15	0.25	0.13	<0.05

Variance estimates:

	Estimate	l-95% CI	u-95% CI	Mean rand. est.	SD rand. est.	p-value
Intercept	-0.20	-0.25	-0.16	0.30	0.02	<0.05
Prop. missing	0.08	0.04	0.12	0.01	0.02	<0.05
Sociality	-0.09	-0.13	-0.06	0.00	0.02	<0.05
Prop. missing : Sociality	0.10	0.06	0.14	0.00	0.02	<0.05

Supplementary table 3: Model results for the difference in individuals' betweenness centrality in survivor-only networks (Figure 2 a, c to f) from the survivor-only pre-event network (Figure 2 b). Explanatory variables are an individual's level of association with missing individuals, pre-event sociality(weighted degree in the full pre-event network), timestep and the interaction between them. p values obtained by comparisons with randomised versions of the survivor-only post-event networks.

	Estimate	l-95% CI	u-95% CI	Mean rand. est.	SD rand. est.	p-value
Intercept	35.78	-224.32	289.52	37.31	37.85	0.47
Timestep 1 (-10 to -6 days)	-157.36	-541.37	223.73	-131.56	165.57	0.36
Timestep 3 (1 to 5 days)	-184.23	-562.21	204.71	-161.29	50.54	0.41
Timestep 4 (6 to 10 days)	11.84	-380.48	402.30	12.91	49.33	0.49
Timestep 5 (11 to 15 days)	-30.10	-410.74	369.90	-8.10	120.85	0.29
Timestep 6 (16 to 20 days)	-5.67	-381.34	385.07	31.27	45.17	<0.05
Prop. missing	-3.26	-365.37	368.54	-3.68	24.54	0.5
Sociality	19.81	-349.42	385.13	9.54	13.00	<0.05
Timestep 1 (-10 to -6 days) : Prop. missing	18.27	-382.44	419.39	-52.53	117.11	0.27
Timestep 3 (1 to 5 days) : Prop. missing	133.71	-270.29	532.81	-39.52	105.38	<0.05
Timestep 4 (6 to 10 days) : Prop. missing	147.26	-264.95	555.12	-68.04	157.77	<0.05
Timestep 5 (11 to 15 days) : Prop. missing	-49.72	-463.63	361.53	-85.92	140.87	0.39
Timestep 6 (16 to 20 days) : Prop. missing	78.84	-323.83	486.73	-87.94	108.27	<0.05
Timestep 1 (-10 to -6 days) : Sociality	44.94	-329.76	413.55	160.64	111.53	<0.05
Timestep 3 (1 to 5 days) : Sociality	252.14	-120.66	613.31	230.03	128.26	0.42
Timestep 4 (6 to 10 days) : Sociality	-160.25	-536.16	208.22	115.09	146.84	<0.05
Timestep 5 (11 to 15 days) : Sociality	345.54	-29.89	720.12	172.22	148.02	<0.05
Timestep 6 (16 to 20 days) : Sociality	-241.76	-615.23	134.53	175.52	177.98	<0.05
Prop. missing : Sociality	-7.42	-413.46	393.33	-0.40	28.91	0.4
Timestep 1 (-10 to -6 days) : Prop. missing : Sociality	-205.50	-634.58	222.37	-63.63	115.04	<0.05
Timestep 3 (1 to 5 days) : Prop. missing : Sociality	83.31	-352.62	534.73	-9.52	126.92	<0.05
Timestep 4 (6 to 10 days) : Prop. missing : Sociality	-137.53	-571.50	301.22	-46.43	134.99	0.24
Timestep 5 (11 to 15 days) : Prop. missing : Sociality	-242.65	-688.59	183.43	-78.99	151.94	<0.05
Timestep 6 (16 to 20 days) : Prop. missing : Sociality	-109.48	-540.79	312.08	27.59	110.94	<0.05

Variance estimates:

	Estimate	l-95% CI	u-95% CI	Mean rand. est.	SD rand. est.	p-value
Intercept	7.65	7.61	7.70	7.69	0.05	0.26
Prop. missing	0.02	-0.03	0.07	-0.02	0.05	0.23
Sociality	-0.08	-0.12	-0.04	-0.04	0.05	0.22
Prop. missing : Sociality	-0.02	-0.07	0.02	0.00	0.06	0.38

Supplementary table 4: Model results for the difference in individuals' degree centrality in survivor-only networks (Figure 2 a, c to f) from the survivor-only pre-event network (Figure 2 b). Explanatory variables are an individual's level of association with missing individuals, pre-event sociality(weighted degree in the full pre-event network), timestep and the interaction between association with missign individuals and pre-event sociality. p values obtained by comparisons with randomised versions of the survivor-only post-event networks.

	Estimate	l-95% CI	u-95% CI	Mean rand. est.	SD rand. est.	p-value
Intercept	-0.26	-1.02	0.47	-0.38	0.19	0.28
Timestep 1 (-10 to -6 days)	-0.31	-1.18	0.54	-1.45	1.00	<0.05
Timestep 3 (1 to 5 days)	5.18	4.31	6.05	5.57	0.70	0.28
Timestep 4 (6 to 10 days)	0.17	-0.71	1.04	-1.22	0.42	<0.05
Timestep 5 (11 to 15 days)	-1.78	-2.64	-0.90	-2.47	0.31	<0.05
Timestep 6 (16 to 20 days)	-1.82	-2.69	-0.93	-2.31	0.24	<0.05
Prop. missing	-0.32	-0.86	0.23	0.96	0.28	<0.05
Sociality	-0.62	-1.10	-0.15	-2.62	0.26	<0.05
Prop. missing : Sociality	-0.53	-1.11	0.04	-1.91	0.25	<0.05

Variance estimates:

	Estimate	l-95% CI	u-95% CI	Mean rand. est.	SD rand. est.	p-value
Intercept	1.64	1.60	1.68	2.35	0.03	<0.05
Prop. missing	0.16	0.12	0.21	0.04	0.03	<0.05
Sociality	0.04	0.00	0.08	0.01	0.03	<0.05
Prop. missing : Sociality	0.17	0.12	0.21	0.03	0.03	<0.05

Supplementary table 5: Model results for the difference in individuals' weighted degree centrality in survivor-only networks (Figure 2 a, c to f) from the survivor-only pre-event network (Figure 2 b). Explanatory variables are an individual's level of association with missing individuals, pre-event sociality(weighted degree in the full pre-event network), timestep and the interaction between association with missign individuals and pre-event sociality. p values obtained by comparisons with randomised versions of the survivor-only post-event networks.

	Estimate	l-95% CI	u-95% CI	Mean rand. est.	SD rand. est.	p-value
Intercept	0.06	-0.07	0.18	0.05	0.03	0.46
Timestep 1 (-10 to -6 days)	-0.91	-1.06	-0.76	-1.16	0.07	<0.05
Timestep 3 (1 to 5 days)	0.05	-0.10	0.20	-0.31	0.08	<0.05
Timestep 4 (6 to 10 days)	-0.39	-0.54	-0.25	-0.71	0.07	<0.05
Timestep 5 (11 to 15 days)	0.40	0.26	0.54	0.30	0.06	<0.05
Timestep 6 (16 to 20 days)	0.01	-0.14	0.14	0.00	0.05	0.47
Prop. missing	0.07	-0.02	0.16	0.70	0.04	<0.05
Sociality	-0.28	-0.36	-0.19	-0.85	0.04	<0.05
Prop. missing : Sociality	-0.01	-0.10	0.09	0.21	0.04	<0.05

Variance estimates:

	Estimate	l-95% CI	u-95% CI	Mean rand. est.	SD rand. est.	p-value
Intercept	-0.18	-0.22	-0.14	0.37	0.02	<0.05
Prop. missing	0.08	0.04	0.12	0.03	0.02	<0.05
Sociality	-0.09	-0.13	-0.06	0.01	0.02	<0.05
Prop. missing : Sociality	0.10	0.06	0.14	-0.02	0.01	<0.05

Supplementary table 6: Model results for the difference in individuals' betweenness centrality in survivor-only networks (Figure 2 a, c to f) from the survivor-only pre-event network (Figure 2 b). Explanatory variables are an individual's level of association with missing individuals, pre-event sociality(weighted degree in the full pre-event network), timestep and the interaction between association with missign individuals and pre-event sociality. p values obtained by comparisons with randomised versions of the survivor-only post-event networks.

	Estimate	l-95% CI	u-95% CI	Mean rand. est.	SD rand. est.	p-value
Intercept	29.95	-208.75	264.49	36.19	37.33	0.48
Timestep 1 (-10 to -6 days)	-127.75	-493.73	229.62	-120.98	149.61	0.43
Timestep 3 (1 to 5 days)	-156.20	-523.15	218.01	-154.74	50.84	0.47
Timestep 4 (6 to 10 days)	-12.51	-371.40	361.59	6.06	47.05	0.35
Timestep 5 (11 to 15 days)	57.42	-296.27	429.30	6.29	139.90	0.25
Timestep 6 (16 to 20 days)	-39.30	-402.61	316.81	26.27	49.46	<0.05
Prop. missing	10.33	-263.00	290.43	-58.32	48.02	<0.05
Sociality	35.63	-226.76	299.61	155.01	48.53	<0.05
Prop. missing : Sociality	-90.16	-375.43	197.70	-16.56	49.73	<0.05

Variance estimates:

	Estimate	l-95% CI	u-95% CI	Mean rand. est.	SD rand. est.	p-value
Intercept	7.65	7.61	7.69	7.69	0.05	0.2
Prop. missing	0.00	-0.05	0.05	-0.03	0.06	0.31
Sociality	-0.08	-0.11	-0.04	-0.04	0.05	0.24
Prop. missing : Sociality	-0.06	-0.11	-0.02	0.00	0.07	<0.05

Supplementary table 7: Model results for the difference in individuals' degree centrality in survivor-only networks (Figure 2 a, c to f) from the survivor-only pre-event network (Figure 2 b). Explanatory variables are an individual's level of association with missing individuals, sex, timestep and the interaction between them. p values obtained by comparisons with randomised versions of the survivor-only post-event networks.

	Estimate	l-95% CI	u-95% CI	Mean rand. est.	SD rand. est.	p-value
Intercept	0.04	-1.00	1.08	0.01	0.04	<0.05
Timestep 1 (-10 to -6 days)	-0.05	-1.30	1.23	-2.16	1.21	<0.05
Timestep 3 (1 to 5 days)	5.37	4.11	6.62	4.84	1.18	0.33
Timestep 4 (6 to 10 days)	0.04	-1.21	1.28	-2.13	0.60	<0.05
Timestep 5 (11 to 15 days)	-1.80	-3.08	-0.56	-3.43	0.48	<0.05
Timestep 6 (16 to 20 days)	-1.79	-3.06	-0.53	-3.23	0.41	<0.05
Prop. missing	0.07	-1.08	1.24	-0.01	0.18	0.31
Male	-0.04	-1.60	1.50	0.00	0.06	0.22
Timestep 1 (-10 to -6 days) : Prop. missing	-0.28	-1.75	1.18	2.41	1.00	<0.05
Timestep 3 (1 to 5 days) : Prop. missing	0.54	-0.98	2.01	1.90	0.98	<0.05
Timestep 4 (6 to 10 days) : Prop. missing	0.27	-1.21	1.72	2.27	0.89	<0.05
Timestep 5 (11 to 15 days) : Prop. missing	-0.55	-2.03	0.93	2.36	0.69	<0.05
Timestep 6 (16 to 20 days) : Prop. missing	-1.30	-2.80	0.19	2.24	0.76	<0.05
Timestep 1 (-10 to -6 days) : Male	-0.76	-2.58	1.09	1.39	1.51	<0.05
Timestep 3 (1 to 5 days) : Male	-0.17	-2.04	1.73	1.38	2.28	0.25
Timestep 4 (6 to 10 days) : Male	-0.09	-1.95	1.82	1.68	1.11	<0.05
Timestep 5 (11 to 15 days) : Male	-0.72	-2.59	1.13	1.81	0.98	<0.05
Timestep 6 (16 to 20 days) : Male	-1.32	-3.21	0.56	1.70	0.91	<0.05
Prop. missing : Male	0.26	-1.51	1.98	0.01	0.07	<0.05
Timestep 1 (-10 to -6 days) : Prop. missing : Male	0.13	-2.08	2.37	0.30	1.58	0.44
Timestep 3 (1 to 5 days) : Prop. missing : Male	1.77	-0.41	4.07	0.76	1.76	0.27
Timestep 4 (6 to 10 days) : Prop. missing : Male	0.38	-1.83	2.52	0.56	1.16	0.41
Timestep 5 (11 to 15 days) : Prop. missing : Male	1.48	-0.73	3.70	0.71	0.98	0.23
Timestep 6 (16 to 20 days) : Prop. missing : Male	1.70	-0.50	3.93	0.51	0.93	<0.05

Variance estimates:

	Estimate	l-95% CI	u-95% CI	Mean rand. est.	SD rand. est.	p-value
Intercept	1.63	1.59	1.67	2.34	0.04	<0.05
Prop. missing	0.15	0.10	0.21	0.00	0.03	<0.05

Supplementary table 8: Model results for the difference in individuals' weighted degree centrality in survivor-only networks (Figure 2 a, c to f) from the survivor-only pre-event network (Figure 2 b). Explanatory variables are an individual's level of association with missing individuals, sex, timestep and the interaction between them. p values obtained by comparisons with randomised versions of the survivor-only post-event networks.

	Estimate	l-95% CI	u-95% CI	Mean rand. est.	SD rand. est.	p-value
Intercept	0.00	-0.19	0.18	0.00	0.00	0.34
Timestep 1 (-10 to -6 days)	-0.77	-0.97	-0.57	-1.20	0.08	<0.05
Timestep 3 (1 to 5 days)	0.09	-0.11	0.29	-0.37	0.11	<0.05
Timestep 4 (6 to 10 days)	-0.28	-0.50	-0.08	-0.76	0.10	<0.05
Timestep 5 (11 to 15 days)	0.54	0.33	0.75	0.22	0.11	<0.05
Timestep 6 (16 to 20 days)	0.25	0.04	0.45	-0.04	0.09	<0.05
Prop. missing	-0.01	-0.20	0.20	0.00	0.01	0.48
Male	0.01	-0.27	0.26	0.00	0.01	0.46
Timestep 1 (-10 to -6 days) : Prop. missing	0.12	-0.11	0.35	1.03	0.07	<0.05
Timestep 3 (1 to 5 days) : Prop. missing	0.26	0.02	0.49	1.04	0.12	<0.05
Timestep 4 (6 to 10 days) : Prop. missing	0.28	0.06	0.51	1.04	0.10	<0.05
Timestep 5 (11 to 15 days) : Prop. missing	0.17	-0.06	0.40	1.02	0.15	<0.05
Timestep 6 (16 to 20 days) : Prop. missing	0.11	-0.12	0.35	1.04	0.14	<0.05
Timestep 1 (-10 to -6 days) : Male	-0.10	-0.40	0.21	0.16	0.11	<0.05
Timestep 3 (1 to 5 days) : Male	0.18	-0.13	0.48	0.21	0.17	0.31
Timestep 4 (6 to 10 days) : Male	-0.04	-0.35	0.26	0.16	0.16	<0.05
Timestep 5 (11 to 15 days) : Male	-0.20	-0.51	0.11	0.23	0.21	<0.05
Timestep 6 (16 to 20 days) : Male	-0.44	-0.74	-0.13	0.15	0.20	<0.05
Prop. missing : Male	0.02	-0.27	0.31	-0.01	0.01	0.44
Timestep 1 (-10 to -6 days) : Prop. missing : Male	0.04	-0.30	0.37	-0.22	0.11	0.42
Timestep 3 (1 to 5 days) : Prop. missing : Male	0.14	-0.20	0.48	-0.31	0.19	0.41
Timestep 4 (6 to 10 days) : Prop. missing : Male	0.04	-0.29	0.37	-0.24	0.17	0.31
Timestep 5 (11 to 15 days) : Prop. missing : Male	-0.01	-0.35	0.32	-0.20	0.21	0.26
Timestep 6 (16 to 20 days) : Prop. missing : Male	0.01	-0.33	0.35	-0.27	0.24	0.3

Variance estimates:

	Estimate	l-95% CI	u-95% CI	Mean rand. est.	SD rand. est.	p-value
Intercept	-0.19	-0.23	-0.15	0.35	0.02	<0.05
Prop. missing	0.10	0.05	0.14	-0.02	0.02	<0.05

Supplementary table 9: Model results for change in groups size in survivor-only networks (Supplementary figure 3 a, c to f) from the survivor-only pre-event network (Supplementary figure 3 b). Explanatory variables are proportion of missing individuals, group size in the full pre-event network, timestep and the interaction between them.

	Estimate	l-95% CI	u-95% CI
Intercept	0.03	-1.52	1.50
Prop. group missing	0.02	-1.77	1.90
Group Size	-0.02	-1.43	1.37
Timestep 1 (-10 to -6 days)	-0.51	-2.24	1.23
Timestep 3 (1 to 5 days)	0.06	-1.71	1.80
Timestep 4 (6 to 10 days)	0.18	-1.61	1.96
Timestep 5 (11 to 15 days)	-0.74	-2.50	1.05
Timestep 6 (16 to 20 days)	-0.93	-2.70	0.89
Prop. group missing : Group Size	0.05	-2.29	2.53
Prop. group missing : Timestep 1 (-10 to -6 days)	-0.25	-2.39	1.83
Prop. group missing : Timestep 3 (1 to 5 days)	-0.72	-2.96	1.34
Prop. group missing : Timestep 4 (6 to 10 days)	-0.17	-2.33	1.96
Prop. group missing : Timestep 5 (11 to 15 days)	0.00	-2.17	2.15
Prop. group missing : Timestep 6 (16 to 20 days)	0.02	-2.14	2.17
Group Size : Timestep 1 (-10 to -6 days)	-1.01	-2.66	0.61
Group Size : Timestep 3 (1 to 5 days)	-0.13	-1.82	1.49
Group Size : Timestep 4 (6 to 10 days)	0.11	-1.55	1.72
Group Size : Timestep 5 (11 to 15 days)	-1.10	-2.80	0.55
Group Size : Timestep 6 (16 to 20 days)	-1.40	-3.03	0.27
Prop. group missing : Group Size : Timestep 1 (-10 to -6 days)	-0.27	-3.24	2.61
Prop. group missing : Group Size : Timestep 3 (1 to 5 days)	0.32	-2.55	3.20
Prop. group missing : Group Size : Timestep 4 (6 to 10 days)	0.92	-1.95	3.81
Prop. group missing : Group Size : Timestep 5 (11 to 15 days)	1.48	-1.27	4.42
Prop. group missing : Group Size : Timestep 6 (16 to 20 days)	1.65	-1.23	4.57

Supplementary table 10: Model results for change in within group edge density in survivor-only networks (Supplementary figure 3 a, c to f) from the survivor-only pre-event network (Supplementary figure 3 b). Explanatory variables are proportion of missing individuals, group size in the full pre-event network, timestep and the interaction between them.

	Estimate	l-95% CI	u-95% CI
Intercept	0.00	-0.04	0.04
Prop. group missing	0.00	-0.05	0.05
Group Size	0.00	-0.03	0.03
Timestep 1 (-10 to -6 days)	0.00	-0.04	0.04
Timestep 3 (1 to 5 days)	0.01	-0.03	0.05
Timestep 4 (6 to 10 days)	-0.03	-0.07	0.01
Timestep 5 (11 to 15 days)	-0.04	-0.08	0.00
Timestep 6 (16 to 20 days)	-0.05	-0.10	-0.01
Prop. group missing : Group Size	0.00	-0.06	0.06
Prop. group missing : Timestep 1 (-10 to -6 days)	-0.01	-0.07	0.05
Prop. group missing : Timestep 3 (1 to 5 days)	0.01	-0.05	0.06
Prop. group missing : Timestep 4 (6 to 10 days)	-0.03	-0.09	0.03
Prop. group missing : Timestep 5 (11 to 15 days)	-0.02	-0.08	0.03
Prop. group missing : Timestep 6 (16 to 20 days)	-0.04	-0.09	0.02
Group Size : Timestep 1 (-10 to -6 days)	0.01	-0.03	0.05
Group Size : Timestep 3 (1 to 5 days)	0.01	-0.03	0.05
Group Size : Timestep 4 (6 to 10 days)	0.00	-0.03	0.04
Group Size : Timestep 5 (11 to 15 days)	0.01	-0.02	0.05
Group Size : Timestep 6 (16 to 20 days)	0.02	-0.02	0.06
Prop. group missing : Group Size : Timestep 1 (-10 to -6 days)	-0.01	-0.08	0.05
Prop. group missing : Group Size : Timestep 3 (1 to 5 days)	-0.01	-0.07	0.06
Prop. group missing : Group Size : Timestep 4 (6 to 10 days)	-0.05	-0.12	0.01
Prop. group missing : Group Size : Timestep 5 (11 to 15 days)	-0.06	-0.13	0.00
Prop. group missing : Group Size : Timestep 6 (16 to 20 days)	-0.04	-0.11	0.03

Supplementary table 11: Model results for change in groups size in survivor-only networks (Supplementary figure 3 a, c to f) from the survivor-only pre-event network (Supplementary figure 3 b). Explanatory variables are proportion of missing individuals, group size in the full pre-event network, timestep and the interaction between proportion of missing individuals and pre-event group size.

	Estimate	l-95% CI	u-95% CI
Intercept	0.22	-1.12	1.60
Timestep 1 (-10 to -6 days)	-0.43	-1.92	1.10
Timestep 3 (1 to 5 days)	-0.02	-1.57	1.55
Timestep 4 (6 to 10 days)	-0.07	-1.61	1.49
Timestep 5 (11 to 15 days)	-1.13	-2.67	0.40
Timestep 6 (16 to 20 days)	-1.36	-2.87	0.21
Prop. group missing	-0.18	-1.34	0.99
Group Size	-0.62	-1.58	0.28
Prop. group missing : Group Size	0.72	-0.93	2.35

Supplementary table 12: Model results for change in within group edge density in survivor-only networks (Supplementary figure 3 a, c to f) from the survivor-only pre-event network (Supplementary figure 3 b). Explanatory variables are proportion of missing individuals, group size in the full pre-event network, timestep and the interaction between them.

	Estimate	l-95% CI	u-95% CI
Intercept	-0.01	-0.04	0.02
Timestep 1 (-10 to -6 days)	0.00	-0.03	0.03
Timestep 3 (1 to 5 days)	0.01	-0.03	0.04
Timestep 4 (6 to 10 days)	-0.01	-0.05	0.02
Timestep 5 (11 to 15 days)	-0.02	-0.06	0.01
Timestep 6 (16 to 20 days)	-0.04	-0.07	0.00
Prop. group missing	-0.02	-0.05	0.01
Group Size	0.01	-0.01	0.03
Prop. group missing : Group Size	-0.03	-0.07	0.01

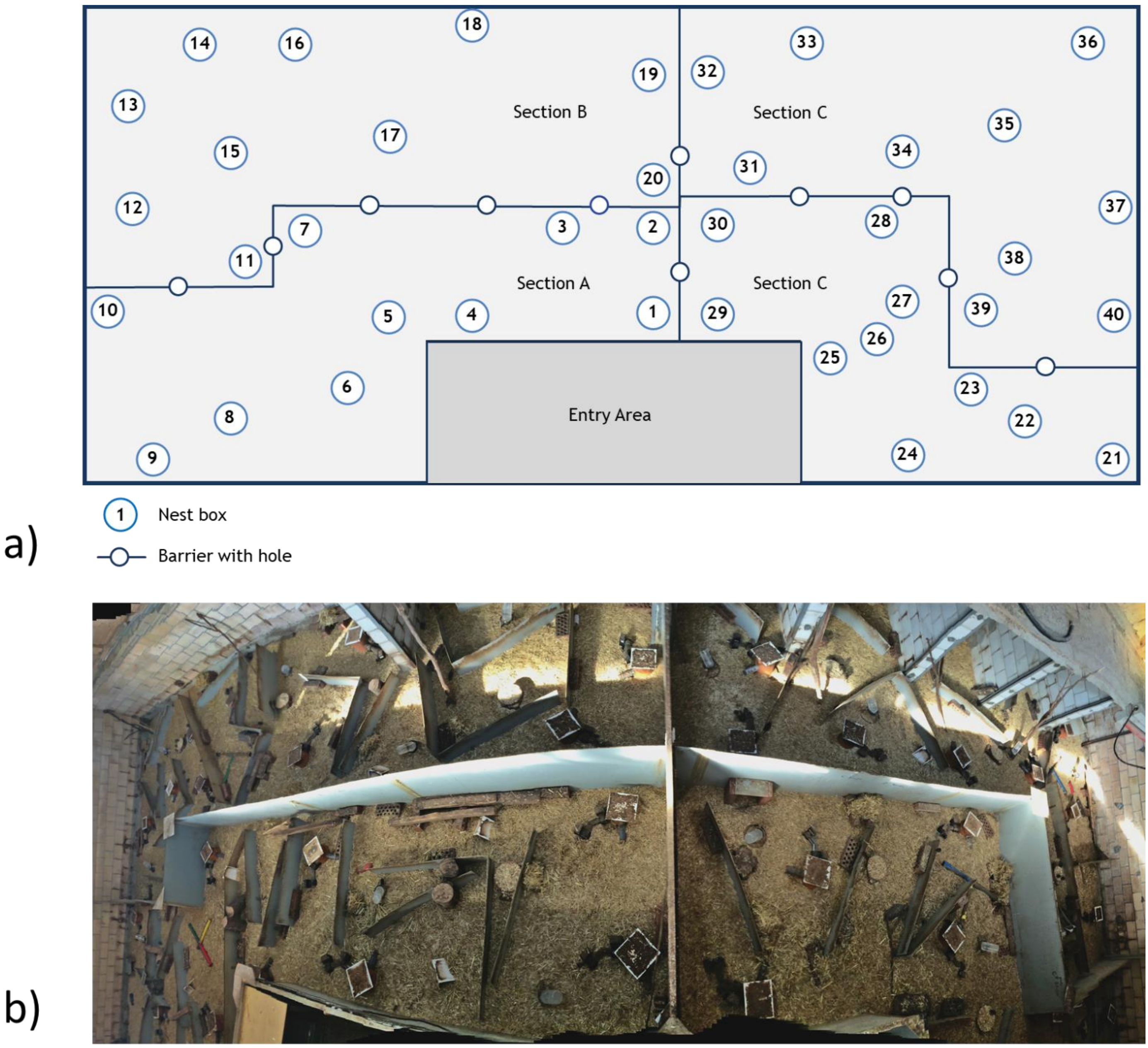

Supplementary figure 1: Barn layout a) Map of the barn indicating location of RFID equipped nest boxes and barriers in separating the four sections, which mice can access through various holes in the barriers. Each section also includes 3 food bowls, 5 water bottles and a number of extra shelters and smaller barriers. b) Panoramic photo of the barn taken from the entry area showing extra shelters and smaller barriers within section.

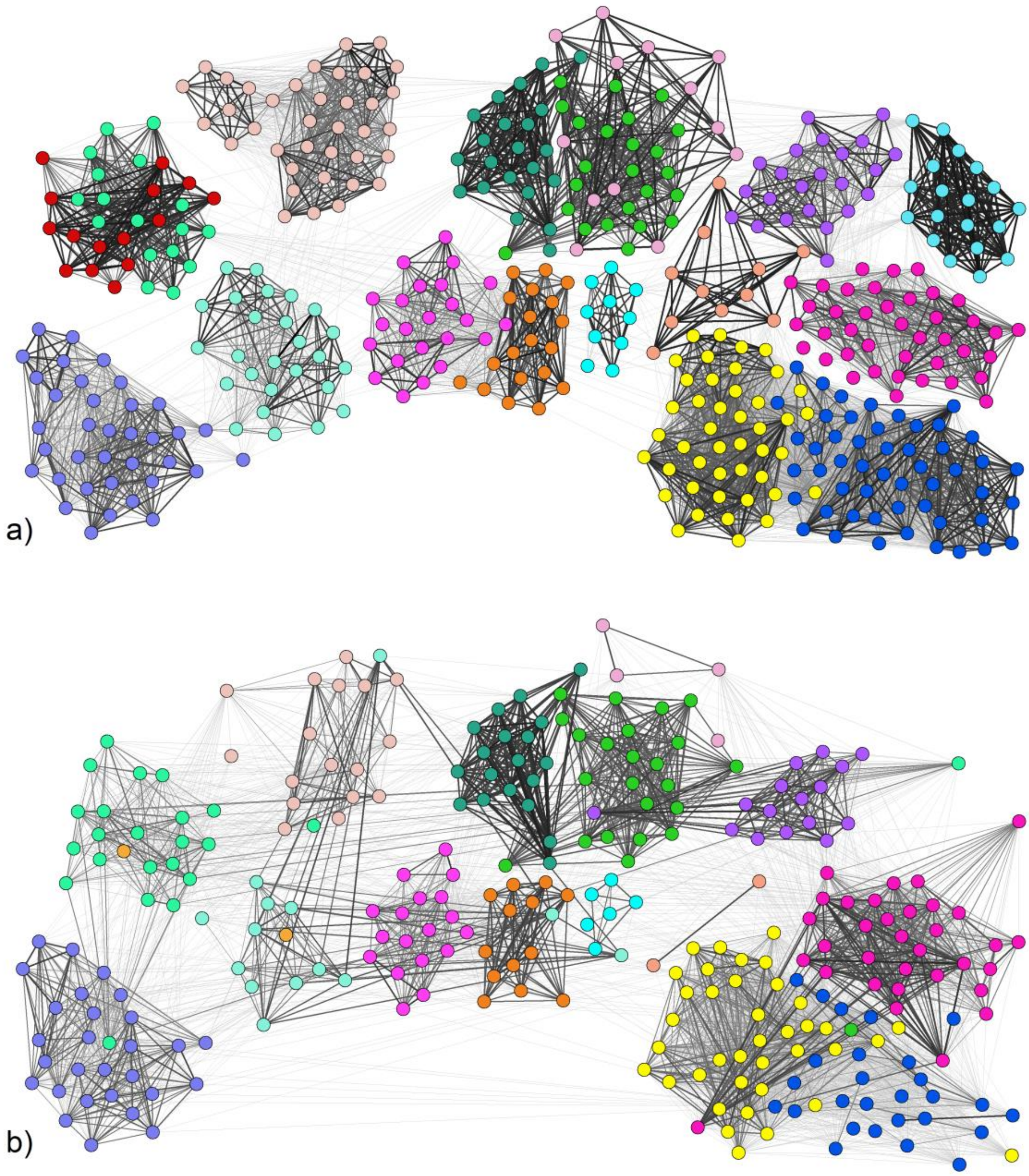

Supplementary figure 2: Full network in the timestep a) 5-1 days immediately before the predation event (timestep 2), and b) 1-5 days immediately after the predation event (timestep 3). Thicker/darker lines denote stronger social associations. Colour of node represents social group as assigned by dynamic community detection (See Figure 3). Node position is approximately based on individuals nestbox use prior to the predation event.

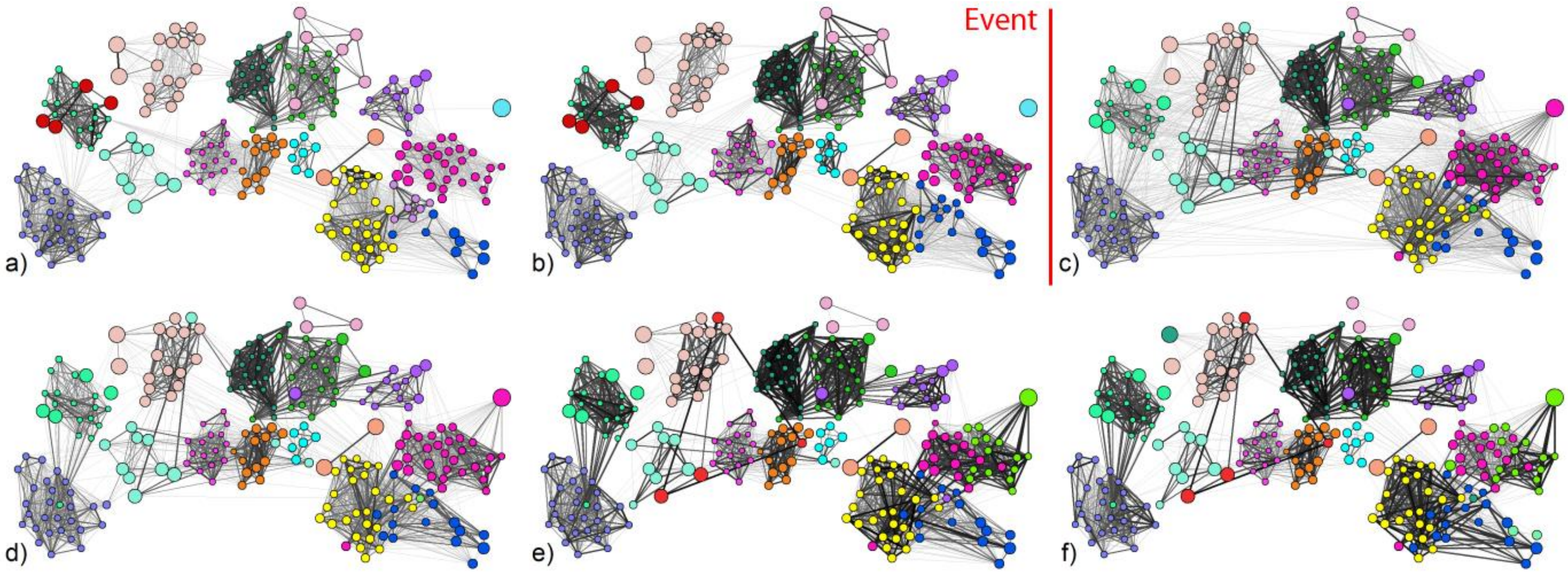

Supplementary figure 3: Within survivor networks a) 2 timesteps before predation event, b) 1 timestep before predation event, c) 1 timestep after predation event, d) 2 timesteps after predation event, e) 3 timesteps after predation event and f) 4 timesteps after predation event. Thickness of edges denotes strength of social connection. Colour of node represents social group as assigned by dynamic community detection (corresponding to the colours in Supplementary figure 4). Size of node indicates the proportion of association lost due to individuals going missing. Figure represents survivor-only networks, though dynamic communities were calculated using full unrestricted networks. Node position is approximately based on individuals nestbox use prior to the predation event.

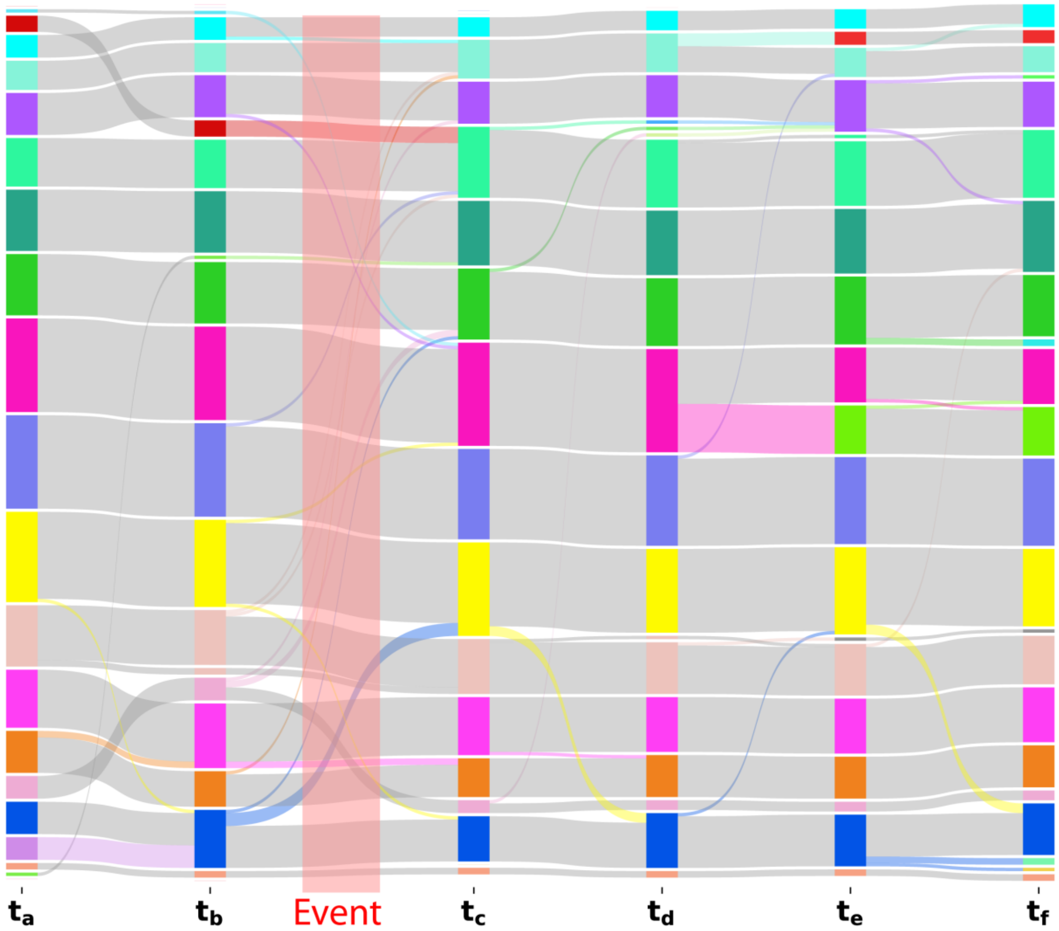

Supplementary figure 4: Alluvial plot illustrating the movements between dynamic communities present in survivor-only networks at a) 2 timesteps before predation event, b) 1 timestep before predation event, c) 1 timestep after predation event, d) 2 timesteps after predation event, e) 3 timesteps after predation event and f) 4 timesteps after predation event. Movements from one community to another are highlighted in the same colour as the source community. Figure represents survivor-only networks, though dynamic communities were calculated using full unrestricted networks.

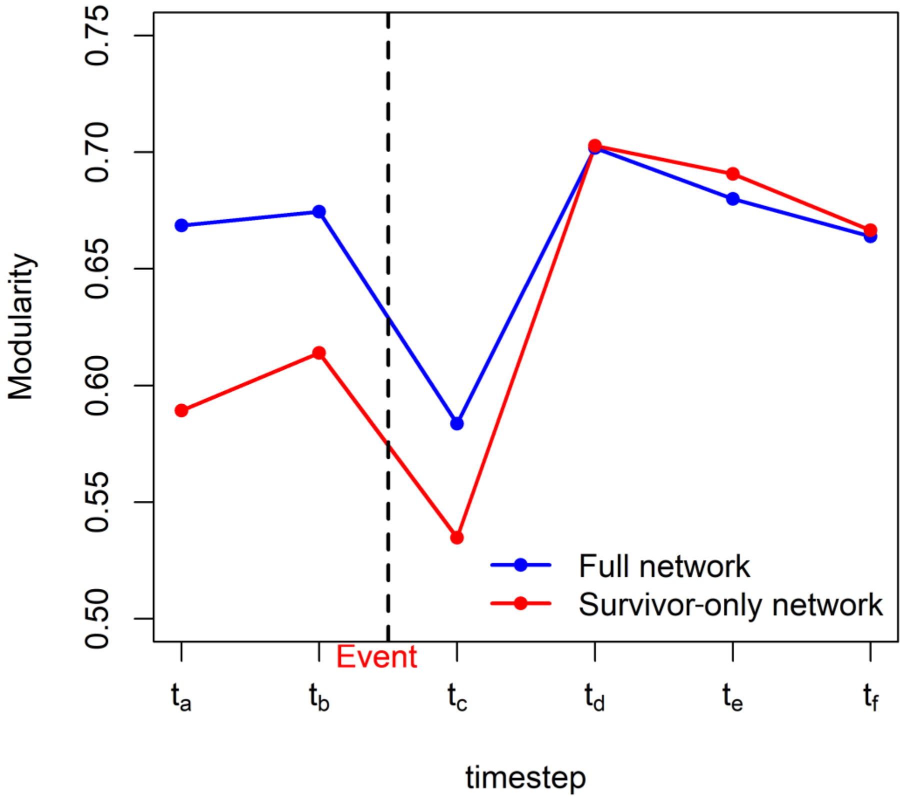

Supplementary figure 5: Network modularity at a) 2 timesteps before predation event, b) 1 timestep before predation event, c) 1 timestep after predation event, d) 2 timesteps after predation event, e) 3 timesteps after predation event and f) 4 timesteps after predation event. Blue line indicates the modularity of the full unrestricted network while red line represents network made up only of those individuals present in all timesteps.
